# Lived experience response to disinformation Campaigns

**DOI:** 10.1371/journal.pmen.0000465

**Published:** 2025-10-16

**Authors:** Parth Sharma, Anastasia Smith, Anoushka Virk, Elza Berk, Geetika Sawhney, Julieann Cullen, Karen Coelho, Matthew Jackman, Meghna Prakash, Preethi Shanmugapriya, Sarah Huang, Shruti Bora, Taylor Locke

**Affiliations:** 1 Sangath, and Global Mental Health Peer Network, Bhopal, India,; 2 Global Mental Health Peer Network, Johannesburg, South Africa; 3 Sangath, Bhopal, India; 4 Therapy Now Global, Cape Town, South Africa; 5 Global Mental Health Peer Network, Bangalore, India; 6 Global Mental Health Peer Network, Dublin, Ireland; 7 TISS, Mumbai, India; 8 Centre for Disability Research and Policy, University of Sydney, Australia; 9 Soft Spaces Therapy, Delhi, India; 10 Inclusive Wellbeing, Milir: Anticaste MentalHealth Collective, Tamil Nadu, India; 11 Global Mental Health Peer Network, Manila, Philippines; 12 Generation Mental Health, Goa, India; 13 Global Rights Alliance, Global Mental Health Peer Network, New York, United States of America; PLOS: Public Library of Science, UNITED KINGDOM OF GREAT BRITAIN AND NORTHERN IRELAND

*“This claim [use of Tylenol during pregnancy causes Autism] embodies the scapegoat archetype, where society seeks someone to blame for what it doesn’t understand. Instead of embracing neurodiversity as a natural part of human variation, people direct their fears toward pregnant individuals and medical interventions. These claims reopen wounds for every neurodivergent person who’s ever been told that they shouldn’t exist”*

*-Elza Berk, Person with Lived Experience, South Africa*

## Understanding Disinformation

Disinformation campaigns are deliberately designed to spread misleading and incorrect information as propaganda tools to influence the general public’s opinion. Especially in the context of Health Communication, disinformation involves omission of scientific knowledge and often exaggerated claims that give people a false impression about their health and treatment options [1].

When US President Donald Trump, in a White House Press Conference on September 22, 2025, advised pregnant women to not take paracetamol (acetaminophen sold as Tylenol in the USA), it contributed to widespread disinformation linking Tylenol use to Autism in children [2]. This comment was not made in isolation and was echoed by US Health Secretary Robert F Kennedy Junior who has, on several occasions, linked vaccination with autism and mental health conditions [3]. The US Food and Drug Administration (FDA) later announced that it would attach a warning label on the drug, citing ‘possible association’ between Autism and the use of acetaminophen during pregnancy [4].

Experts have dispelled this claim as unverified and have urged people to exercise caution as they consume information about treatment options [5]. However, this recent example of the growing culture of disinformation around neurodiversity and mental health begs the question: what are the consequences of such campaigns on people with lived experience?

## Disinformation in the digital age

Digital technologies have allowed immense progress in disseminating information about mental health, however an exponential increase in use of social media platforms as a source of knowledge sharing has also come with widespread online disinformation. International organizations such as the World Health Organisation and the World Economic Forum call the rising culture of disinformation a direct risk to democracy, given its potential to dismiss the democratic process and its role in creating social unrest [6,7].

While one can argue that statements about mental health made in one country by some leaders may not influence health policy in another, they do however risk reinforcing existing prejudices against people with neurodivergent or mental health conditions. With recent developments in AI in communications, disinformation is harder to track and often leads to fueling political tensions [8].

It is also a public health issue placing people in strategically underserved communities at greater risk of receiving wrong medical guidance. The Covid-19 pandemic was a glaring example of such cases [9].

When global leaders make unsupported claims about health it creates a ripple effect on digital platforms that further sanctions the spread of misleading information and discrimination against people in need of understanding and threatens their well-being. This erodes trust in medical guidance, which risks the reduced collective capacity to respond to health emergencies in the future. A concern highlighted across this year’s theme for World Mental Health Day 2025.

## Stigma and Discrimination

Fabrications, omissions, or exaggerations of research around Autism act as fodder for the larger public and add to the prejudice of viewing neurodivergence and mental health, and, in extension, disabilities, as an individual deficit that requires correction. This perpetuates structural discrimination and violence against people with autism who repeatedly experience other-ing due to the negative public perception around neurodiversity [10].

Disinformation not only produces a public spectacle; it overlaps with many profound, intersecting consequences, which inevitably achieve their desired outcome of increased stigma, discrimination and hostility towards affected communities. From increased skepticism and uncertainty around seeking mental health support to the evident polarization of public opinion on medications and treatment options, disinformation campaigns thrive on manipulating public emotions. This can often have consequences that may extend beyond individual attitudes to systemic harms including repressive policy shifts that defund services that disproportionately impact neurodivergent or disabled communities [11].

By linking paracetamol (acetaminophen, sold as Tylenol in the USA) and its use in pregnancy to autism in children, pregnant individuals were disproportionately placed at the center of fearmongering. Not only are women further subjected to media scrutiny, but their reproductive rights are also being called into question. For women already at the margins devoid of affordable and accessible healthcare, especially those from racial, ethnic, religious, caste, and class minorities, this patriarchal blame further endangers their well-being and reduces their autonomy over their own body. This narrative also weaponises Autism and in extension Mental Health to move towards ‘finding a cure’, positioning neurodivergent people as pathological and in need of medical correction, further perpetuating ableism.

## Eugenics and the “*Epidemic of Autism*”

Disinformation plays a significant role in polarising the public. As people become more divided over a subject such as autism, there lies a risk that individuals may reinforce their pre-existing prejudices about autism and become selective in their acceptance of scientific evidence or lived experience. Not only does disinformation then sanction prejudice, it perpetuates it through leaders and governing bodies.

The very notion that neurodiversity is preventable creates a social campaign where the focus moves from providing accommodations to neurodivergent people as a matter of policy, to *prevention* of a pathology. This approach risks leading to a skewed resource distribution towards ‘finding a cure’, and more often than not those that are neurodivergent are villanised and viewed as ill to justify this shift. This rhetoric is reminiscent of American Eugenics and echoes an increasingly authoritarian view of health which is predicated on viewing people to only have value if they fit a narrow neo-colonial lens of productivity and capital [12]. We are of the view that statements made by the Trump Administration on sending neurodivergent people to wellness farms to be “re-parented over the course of years” in light of the “epidemic of autism”, draw alarming parallels to prior human rights violations across history [13].

When authoritarian leaders scapegoat neurodivergence, they tap into existing ableist structures, redirect economic anxieties onto vulnerable populations, position themselves as defenders against “preventable tragedies,” and promote the necropolitical ideology that some lives are inherently less human than others. Amid the overwhelming noise of disinformation, it is now more imperative than ever to center lived experience and humanize neurodiversity in decision-making, research, practice, and policy, and push back against such authoritarianism and biopolitics.

## Lived Experience Response to Disinformation Campaigns

As individuals with intersectional lived experience from across the world, we propose three key strategies to address disinformation campaigns. We recognise and acknowledge that these recommendations do not represent a monolithic perspective on Lived Experience and Neurodivergence and offer one of many approaches to address disinformation moving forward.

**Promote Media Literacy:** Disinformation thrives on creating a spectacle out of facts. Through media literacy campaigns parents, caregivers and educators can critically engage with sources, verify claims made by people in positions of power and make informed choices about supporting platforms [14]. However this would not be possible without intentional efforts to ensure that the public has open access to relevant research and information. A space where researchers work in collaboration with people with lived experience to disseminate information.

**Collective Un-Learning**: Disinformation demands a society that is divided over the authenticity of scientific facts. This allows further polarisation of people and generates unrest. Community spaces are well positioned to act as watchdogs over false claims, and serve as platforms for plain language dissemination of expert evidence [15]. This culture of “collective un-learning” requires democratization of knowledge production and will allow people to engage in critical thinking within their communities through embedded research.

**Centering Lived Experience**: At the centre of Disinformation lies fabricated narratives about people in need of care and support. By centering Intersectional Lived Experience and creating intentional spaces for neurodivergent people to share their truth as they see fit, we can tackle the increasingly polarizing nature of Disinformation and help reduce stigma [16].

To address the growing culture of disinformation, now more than ever it is necessary that people with lived experience are key decision makers in research, practice and policy.
